# 

**Published:** 1890-02

**Authors:** 


					﻿OLD SERIES
Vol. xxv
<7 1855 4
NEWSfRwO,
v°i. vr ybc.12.
«fe IHmsSE
(sTnURNAIi^A^,

1W. F. WESTMORELAND, M.D.
H. V. M. MILLER, M.D., LL.D.
VIRGIL 0. HARDON, M.D.
F. W. McRAE, M.D., M’n’g’ng Ed.
Mjbushed By
JAMES P.HARRISON&C®
•Atlanta ,ga. JM
SUBSCRIPTION t 12.50 A YEAR
				

